# Management of severe asthma exacerbation: guidelines from the Société Française de Médecine d’Urgence, the Société de Réanimation de Langue Française and the French Group for Pediatric Intensive Care and Emergencies

**DOI:** 10.1186/s13613-019-0584-x

**Published:** 2019-10-10

**Authors:** Philippe Le Conte, Nicolas Terzi, Guillaume Mortamet, Fekri Abroug, Guillaume Carteaux, Céline Charasse, Anthony Chauvin, Xavier Combes, Stéphane Dauger, Alexandre Demoule, Thibaut Desmettre, Stephan Ehrmann, Bénédicte Gaillard-Le Roux, Valérie Hamel, Boris Jung, Sabrina Kepka, Erwan L’Her, Mikaël Martinez, Christophe Milési, Élise Morawiec, Mathieu Oberlin, Patrick Plaisance, Robin Pouyau, Chantal Raherison, Patrick Ray, Mathieu Schmidt, Arnaud W. Thille, Jennifer Truchot, Guillaume Valdenaire, Julien Vaux, Damien Viglino, Guillaume Voiriot, Bénédicte Vrignaud, Sandrine Jean, Eric Mariotte, Pierre-Géraud Claret

**Affiliations:** 1 Service d’Accueil des Urgences, CHU de Nantes, 5 allée de l’île gloriette, 44093 Nantes Cedex 1, France; 2 PHU3, Faculté de Médecine 1, rue Gaston Veil, 44035 Nantes, France; 3 Service de Médecine Intensive Réanimation, CHU de Grenoble Alpes, 38000 Grenoble, France; 4 INSERM, U1042, University of Grenoble-Alpes, HP2, 38000 Grenoble, France; 5 Service de Réanimation Pédiatrique, CHU de Grenoble Alpes, 38000 Grenoble, France; 6 Service de réanimation, CHU de Monastir, Monastir, Tunisia; 7 Service de MIR, Hôpital Henri Mondor, APHP, Créteil, France; 8 Pediatric Emergency Department, CHU Pellegrin Enfants, Bordeaux, France; 9 Service des Urgences, Hôpital Lariboisière, APHP, Paris, France; 10 Service des Urgences, CHU de la Réunion, Saint-Denis, France; 11 Pediatric Intensive Care Unit, Robert Debré Hospital, APHP, Paris, France; 12 Groupe Hospitalier Pitié-Salpêtrière Charles Foix, Service de Pneumologie, Médecine Intensive et Réanimation (Département R3S), AP-HP, INSERM, UMRS1158 neurophysiologie respiratoire expérimentale et clinique, Sorbonne Université, Paris, France; 13 Service des Urgences, CHU de Besançon, Besançon, France; 14 Médecine Intensive Réanimation, INSERM CIC 1415, réseau CRICS-TriggerSEP, CHRU de Tours and Centre d’Etude des Pathologies Respiratoires, INSERM U1100, faculté de médecine, Université de Tours, Tours, France; 15 Pediatric Intensive Care Unit, Women and Children’s University Hospital, Nantes, France; 16 Service des Urgences, CHU de Toulouse, Toulouse, France; 17 Service de MIR, CHU de Montpelliers, Montpellier, France; 18 Service des Urgences, CHU de Strasbourg, Strasbourg, France; 19 Service de MIR, CHRU de Brest, Brest, France; 20 Pôle Urgences, centre hospitalier du Forez, 42605 Montbrison, France; 21 Réseau d’urgence Ligérien Ardèche Nord (REULIAN), centre hospitalier Le Corbusier, 42700 Firminy, France; 22 Département de Pédiatrie Néonatale et Réanimations, CHU de Montpellier, Montpellier, France; 23 Service de Pneumologie et Réanimation, GH Pitié-Salpêtrière, APHP, Paris, France; 24 Service des Urgences, centre hospitalier de Cahors, Cahors, France; 25 Service des Urgences, AP-HP, hôpital la Riboisière, Paris, France; 26 Pediatric Intensive Care Unit, Women‐Mothers and Children’s University Hospital, Lyon, France; 27 service de Pneumologie, CHU de Bordeaux, Pessac, France; 28 Service des Urgences, CHU de Dijon, faculté de médecine de Dijon, Dijon, France; 29 INSERM, UMRS_1166-ICAN, Institute of Cardiometabolism and Nutrition, Pitié–Salpêtrière Hospital, Medical Intensive Care Unit, Sorbonne Universités, 75651 Paris Cedex 13, France; 30 CHU de Poitiers, Médecine Intensive Réanimation, Poitiers, France; 31 Service des Urgences, CHU de Bordeaux, Pessac, France; 32 SAMU 94, CHU Henri Mondor, AP-HP, Créteil, France; 33 Service des Urgences Adultes, CHU de Grenoble Alpes, 38000 Grenoble, France; 34 Service de réanimation polyvalente, Hôpital Tenon, APHP, Paris, France; 35 Pediatric Emergency Department, Women and Children’, s University Hospital, Nantes, France; 36 Service de Réanimation Pédiatrique, APHP Hôpital Trousseau, 75012 Paris, France; 37 Service de Médecine Intensive Réanimation, APHP Hôpital Saint Louis, 75010 Paris, France; 38 Service d’Accueil des Urgences, CHU de Nîmes, 30029 Nîmes, France

**Keywords:** Asthma, Severe exacerbation, Guidelines, Emergency, Intensive Care, Pediatric

## Abstract

**Background:**

The French Emergency Medicine Society, the French Intensive Care Society and the Pediatric Intensive Care and Emergency Medicine French-Speaking Group edited guidelines on severe asthma exacerbation (SAE) in adult and pediatric patients.

**Results:**

The guidelines were related to 5 areas: diagnosis, pharmacological treatment, oxygen therapy and ventilation, patients triage, specific considerations regarding pregnant women. The literature analysis and formulation of the guidelines were conducted according to the Grade of Recommendation Assessment, Development and Evaluation methodology. An extensive literature research was conducted based on publications indexed in PubMed™ and Cochrane™ databases. Of the 21 formalized guidelines, 4 had a high level of evidence (GRADE 1+/−) and 7 a low level of evidence (GRADE 2+/−). The GRADE method was inapplicable to 10 guidelines, which resulted in expert opinions. A strong agreement was reached for all guidelines.

**Conclusion:**

The conjunct work of 36 experts from 3 scientific societies resulted in 21 formalized recommendations to help improving the emergency and intensive care management of adult and pediatric patients with SAE.

## Introduction

One of the most frequent chronic illnesses, asthma, affects 300 million people worldwide, 30 million of them in Europe [1]. The term “acute asthma attack” is commonly used by patients, but corresponds to no clinical entity and should no longer be used. Asthma exacerbation is defined as an imbalance in the asthmatic disorder and is provoked acutely or subacutely by an external agent or by poor compliance with treatment [1]. Severe asthma exacerbation results from particularly severe bronchospasm and leads to severe obstructive syndrome. However, there is no consensual clinical definition of severe exacerbation [2, 3]. In these guidelines, we have chosen to define severe asthma exacerbation (SAE) as asthma exacerbation that is life-threatening or requires emergency management or both [4].

## Aims of the guidelines

The most recent guidelines by the Société de réanimation de langue française (SRLF) for severe acute asthma in adults date from 2002. In view of therapeutic advances in noninvasive ventilation (NIV) and high-flow oxygen therapy, recent international guidelines [1, 5, 6] and the need to optimize practices [7], it seemed necessary to summarize current data. In this context, the Société française de médecine d’urgence (SFMU) and the SRLF propose these expert guidelines on the management of severe asthma exacerbation.

## Method

These guidelines were drawn up by a group of experts convened by the SFMU and the SRLF. The group’s agenda was defined beforehand. The organizing committee first defined the questions to be addressed with the coordinators and then designated the experts in charge of each question. The questions were formulated according to a Patient Intervention Comparison Outcome (PICO) format after a first meeting of the expert group. The literature was analyzed and the guidelines were formulated using Grade of Recommendation Assessment, Development and Evaluation (GRADE) methodology. A level of proof was defined for each bibliographic reference cited depending on the type of study and could be reassessed in light of the methodological quality of the study. An overall level of proof was determined for each endpoint, taking into account the level of proof of each bibliographic reference, the between-study consistency of the results, the direct or indirect nature of the results, and cost analysis.

A high overall level of proof enabled formulation of a “strong” recommendation (should be done…GRADE 1+, should not be done…GRADE 1−). A moderate, low, or very low overall level of proof led to the drawing up of an “optional” recommendation (should probably be done…GRADE 2+, should probably not be done…GRADE 2−). When the literature was inexistent or insufficient, the question could be the subject of a recommendation in the form of an expert opinion (the experts recommend…). (Table 1) The proposed recommendations were presented and discussed one by one. The aim was not necessarily to reach a single and convergent opinion of the experts on all the proposals, but to define the points of agreement and the points of disagreement or uncertainty. Each expert then reviewed and rated each recommendation using a scale of 1 (complete disagreement) to 9 (complete agreement). The collective rating was done using a GRADE grid. To approve a recommendation regarding a criterion, at least 50% of the experts had to be in agreement and less than 20% in disagreement. For an agreement to be strong, at least 70% of the experts had to be in agreement. In the absence of strong agreement, the recommendations were reformulated and rated again, with a view to reaching a consensus. Only expert opinions that elicited strong agreement were kept.

**Table 1:** Evidence grading and recommendations formulation

	Recommendations formulation according to GRADE	
High level of evidence	Strong recommendation «should be…»	Grade 1+
Intermediate to low level of evidence	Optional recommendation «should probably…»	Grade 2+
No or insufficient available data	Expert opinion «the experts recommend…»	Expert opinion
Intermediate to low level of evidence	Optional recommendation «should probably not…»	Grade 2−
High level of evidence	Strong recommendation «should not be…»	Grade 1−

### Areas of recommendations

Five areas were defined: diagnosis and elements of the diagnosis, pharmacological treatment, methods of oxygen therapy and ventilation, transfer of patients, specific considerations regarding pregnant women. A bibliographic search was conducted using the MEDLINE database via PubMed and the Cochrane database. For inclusion in the analysis, the publications had to be written in English or French. The analysis focused on recent data according to an order of appraisal ranging from meta-analyses to randomized trials to observational studies. A summary of the recommendations is presented in Table 2.

**Table 2:** Summary of recommendations

R1.1 adult—From first contact with patients with asthma exacerbation, the following severity criteria should be sought: history of hospital admission for asthma or need for mechanical ventilation, recent use of oral corticosteroids, considerable or increasing use of beta-2 adrenergic agonists, age > 70 years, difficulty speaking, altered consciousness, shock, respiratory rate > 30 breaths/min, arguments in favor of an underlying pneumonia	Grade 1+
R1.1 pediatric—From first contact with children with asthma exacerbation, the following severity criteria should probably be sought: allergens polysensitization, insufficiently treated or poorly controlled asthma, history of hospitalization for asthma, exposure to passive smoking, and hypoxemia at initial management	Grade 2+
R1.2 adult—In SAE, chest radiography and blood gas measurements (venous or arterial) should probably be done if there is a diagnostic doubt or non-response to treatment	Grade 2+
R1.2 pediatric—The experts suggest that additional examinations are not more effective at diagnosing SAE in children than physical examination alone	Expert opinion
R2.1—Beta-2 adrenergic agonists should not be administered intravenously first line in adult or pediatric patients with SAE even in mechanically ventilated patients	Grade 1−
R2.2—Beta-2 adrenergic agonists should probably be administered by continuous rather than discontinuous nebulization during the first hour in adult and pediatric patients with SAE	Grade 2+
R2.3—Inhaled anticholinergic drugs should be combined with beta-2 adrenergic agonists in adult and pediatric patients with SAE	Grade 1+
R2.4—The experts suggest administering a 0.5-mg dose of ipratropium bromide every 8 h in adults and children over 6 years of age, and a 0.25-mg dose every 8 h in children under 6 years of age	Expert opinion
R2.5 adult—Systemic corticosteroid therapy should be administered early intravenously or orally (1 mg/kg of methylprednisolone equivalent, maximum 80 mg per day) to all adult patients with SAE	Grade 1+
R2.5 pediatric—Systemic corticosteroid therapy should probably be administered early intravenously or orally (2 mg/kg of methylprednisolone equivalent, maximum 80 mg per day) to children with SAE	Grade 2+
R2.6 adult—Magnesium sulfate should probably not be administered routinely to adult patients with SAE	Grade 2−
R2.6 pediatric—Intravenous magnesium sulfate (dose ≥ 20 mg/kg) should be administered routinely to pediatric patients with SAE	Grade 1+
R2.7—Antibiotic therapy should probably not be administered routinely during SAE in adult and pediatric patients. Antibiotic therapy should probably be reserved for cases of suspected bacterial pneumonia, based on usual clinical, radiological, and laboratory signs	Grade 2−
R3.1—Oxygen therapy titrated to a pulse oxygen saturation of 94% to 98% should probably be administered to adult and pediatric patients with SAE	Grade 2+
R3.2 adult—The experts were unable to recommend the use of NIV in SAE. High-flow nasal oxygen therapy has yet to be assessed in this setting	Expert opinion
R3.2 pediatric—The use of NIV in children with SAE should probably be considered when conventional treatments fail	Grade 2−
R3.2 pediatric—The experts are unable to recommend the use of high-flow nasal oxygen in children with SAE	Expert opinion
R3.3—The experts suggest resorting to intubation in adult and pediatric SAE patients if well-conducted medical treatment fails or if the inaugural clinical presentation is severe (altered consciousness, bradypnea). Intubation should be performed using the orotracheal route, after rapid sequence induction including ketamine in first line of hypnotic agent and succinylcholine or rocuronium, by an experienced physician	Expert opinion
R3.4—The experts suggest prevention of lung overdistension by reducing tidal volume, respiratory rate, and positive end-expiratory pressure (PEEP), and by increasing inspiratory flow, in order to limit plateau pressure in mechanically ventilated adult and pediatric patients with SAE	Expert opinion
R3.5 adult—The experts suggest deep sedation—Richmond Agitation-Sedation Scale (RASS) of − 4 to − 5—at the initial phase of invasive mechanical ventilation, as well as neuromuscular blockers in the most severely ill patients. Their modalities are not specific to SAE. The experts are not able to recommend continuous administration of ketamine or halogenated agents	Expert opinion
R3.5 pediatric—Ketamine and halogenated gas should probably not be used for the sedation of mechanically ventilated children with SAE	Grade 2−
R3.6—Helium should probably not be used as carrier gas in nebulizers in adult and pediatric patients with SAE	Grade 2−
R3.7 adult—The experts suggest that aerosols of salbutamol should be administered to spontaneously breathing patients with SAE using a nebulizer. The experts are unable to recommend a particular method of aerosol administration for patients with SAE receiving mechanical ventilation	Expert opinion
R3.7 pediatric—The experts suggest providing a sufficient flow of air or oxygen to ensure the nebulization of inhaled treatments in spontaneously breathing children with SAE. The experts suggest continuing nebulization using specific systems in children with SAE who are mechanically ventilated	Expert opinion
R3.8—In the absence of compelling data in adult and pediatric patients with SAE, the experts suggest discussing with an expert center the use of extracorporeal life support—venovenous ECMO or extracorporeal CO2 removal (ECCO2R)—in the case of respiratory acidosis and/or severe hypoxemia refractory to optimal medical treatment and to well-conducted mechanical ventilation	Expert opinion
R4.1 adult—The experts suggest that the decision to send patients with SAE home should be based on an assessment taking into account the patient’s characteristics, the frequency of exacerbations, the severity of the initial clinical presentation, the response to treatment, including the progression of PEF, and the patient’s ability to be managed at home (referral to the primary care physician)	Expert opinion
R4.1 pediatric—The experts are unable to establish pediatric guidelines regarding the decision to send home children admitted for SAE	Expert opinion
R4.2 adult—The experts suggest that the discharge prescription for patients treated for SAE in the ER should at least include a short-acting beta-2 adrenergic agonist, oral corticosteroid therapy for a short period, and inhaled corticosteroid therapy if it has not been prescribed before	Expert opinion
R4.2 pediatric—The experts are unable to draw up pediatric guidelines regarding the hospital discharge prescription of children admitted for SAE	Expert opinion
R4.3—The experts suggest that admission to intensive care of adult and pediatric patients with SAE should be discussed early, on a case by case basis, because there are no specific criteria on this subject	Expert opinion
R5.1—Pregnant women with SAE should probably be treated in the same way as the general population, by intensifying their controlling therapy upon admission to the emergency room if necessary	Grade 2+

## Results

### First area: diagnosis and elements of the diagnosis

For patients with asthma exacerbation, what severity criteria in medical history and at initial physical examination are associated with an increased risk of mortality and/or intensive care admission?

R1.1 adult—From first contact with patients with asthma exacerbation, the following severity criteria should be sought: history of hospital admission for asthma or need for mechanical ventilation, recent use of oral corticosteroids, considerable or increasing use of beta-2 adrenergic agonists, age > 70 years, difficulty speaking, altered consciousness, shock, respiratory rate > 30 breaths/min, arguments in favor of an underlying pneumonia.

GRADE 1+, STRONG AGREEMENT

#### Rationale

Several studies have tried to identify factors predictive of severe exacerbation in asthma patients. A 2005 meta-analysis [8] reported a significant association between the risk of death (fatal asthma) or the use of mechanical ventilation (near-fatal asthma) and the following factors: hospitalization for asthma exacerbation, in particular admission to intensive care in the previous 12-month period; history of exacerbation that prompted the use of mechanical ventilation; recent or ongoing treatment with oral corticosteroids; and considerable (≥ 1 salbutamol inhaler per month) or increasing use of beta-2 adrenergic agonists [9]. Lack of controller therapy with inhaled corticosteroids [8] and psychosocial factors [10, 11] seem to be associated with a higher risk of severe exacerbation. Published findings are conflicting. There are no data specific to risk evaluation by medical call centers. The abovementioned factors, along with difficulty speaking, are easily identifiable when a patient calls for emergency medical assistance. They are essential to assessment of asthma exacerbation severity and assessment of the risk of unfavorable progression. This should condition not only the medical decision at the call center, but also prehospital management and destination of patients transfer.

The criteria of clinical severity on admission of patients with SAE have mainly been investigated in observational studies [1]. The criteria of poor prognosis used in these studies were death or the need for mechanical ventilation. In patients admitted to intensive care, advanced age, neurological disorders, and tachycardia were clinical features associated with a poor prognosis [12, 13]. Data concerning hospitalized patients were reported by a 2017 Japanese publication [14]. Age (> 70 years), shock, altered consciousness, and infectious lung disease were associated with increased risk of death. Peak expiratory flow (PEF) measured at admission was seldomly studied, but was not associated with a poor prognosis [15, 16].

R1.1 pediatric—From first contact with children with asthma exacerbation, the following severity criteria should probably be sought: allergens polysensitization, insufficiently treated or poorly controlled asthma, history of hospitalization for asthma, exposure to passive smoking, and hypoxemia at initial management.

GRADE 2+, STRONG AGREEMENT

#### Rationale

Only three prospective studies, all of them single-center, described the risk factors for death or admission to pediatric intensive care in case of severe asthma exacerbation in children [17–19]. The risk factors most commonly found are sensitization to multiple allergens, notably food allergens [17], history of hospitalization, especially admission to intensive care, or insufficiently controlled asthma (frequent nocturnal symptoms, frequent use of short-acting beta-2 adrenergic agonists, of oral corticosteroids, or of pediatric emergency services). Exposure to passive smoking and marked hypoxemia at initial management (oxygen saturation < 91% in ambient air) were reported as factors predictive of admission to intensive care in, respectively, three retrospective case–control studies and one retrospective case–control study.

### First area: diagnosis and elements of the diagnosis

Should additional examinations be performed in patients with SAE in an emergency situation?

R1.2 adult—In SAE, chest radiography and blood gas measurements (venous or arterial) should probably be done if there is a diagnostic doubt or non-response to treatment.

GRADE 2+, STRONG AGREEMENT

#### Rationale

A cohort study failed to show any value of routine chest radiography in patients with SAE [20]. However, several guidelines underline its value in eliminating differential diagnoses. Chest radiography is recommended for patients presenting with wheezing/dyspnea and meeting at least one of the following criteria: history of chronic obstructive pulmonary disease, heart disease or thoracic surgery, suspicion of pneumonitis, immunosuppressed state. Arterial blood gas measurement should only be considered for SAE or exacerbations that fail to respond to initial treatment [21]. Normocapnia and hypercapnia are severity criteria of SAE. Measurement of venous blood PCO2 is easier and less painful than arterial sampling, and a PvCO2 value below 45 mmHg excludes arterial hypercapnia [22]. To date, lung ultrasound has not been evaluated in the diagnosis and management of asthma.

R1.2 pediatric—The experts suggest that additional examinations are not more effective at diagnosing SAE in children than physical examination alone.

EXPERT OPINION

#### Rationale

There is no pediatric study that has assessed the value of additional examinations in improving the reliability of the diagnosis of acute asthma in children. A single prospective, descriptive, single-center study has evaluated the value of chest ultrasound in acute asthma in children and reports that certain ultrasound signs may be associated with unfavorable progression [23]. Only chest radiography could be performed in case of clinical sign or medical history suggestive of an alternate diagnosis (no family history of asthma, fever, localized auscultatory abnormality).

### Second area: pharmacological treatment

What are the methods of administration of beta-2 adrenergic agonists in patients with SAE?

R2.1—Beta-2 adrenergic agonists should not be administered intravenously first line in adult or pediatric patients with SAE even in mechanically ventilated patients.

GRADE 1−, STRONG AGREEMENT

R2.2—Beta-2 adrenergic agonists should probably be administered by continuous rather than discontinuous nebulization during the first hour in adult and pediatric patients with SAE.

GRADE 2+, STRONG AGREEMENT

#### Rationale

Numerous studies [20, 24, 25], including a Cochrane meta-analysis [26], have compared continuous and intermittent administrations of inhaled beta-2 adrenergic agonists. Although there are few studies of high methodological quality, at identical doses, continuous administration was associated with a significant decrease in the number of hospitalizations, and with improvements in the ventilatory parameters forced expiratory volume in 1 s (FEV1) and PEF. The observed effect was greater in patients presenting with significant signs of respiratory airways obstruction [25]. This effect seems to be independent of the dose administered since a comparative study of 7.5 vs 2.5 mg/h in continuous nebulization resulted in a comparable improvement [24]. There was no increase in side effects or poor tolerance with continuous administration [26]. In adults, there is no proof of the advantage of using oxygen, compared with air, as the aerosol carrier gas in non-hypoxemic patients. The intravenous route offers no advantage over inhalation as it has been associated with more side effects [27].

There is no pediatric study that has rigorously compared the efficacy of continuous and intermittent nebulization of short-acting beta-2 adrenergic agonists in children with SAE. Several cohort studies have demonstrated that there is no increase in adverse effects (tachycardia, hypokalemia) with continuous versus intermittent nebulization [28, 29].

### Second area: pharmacological treatment

Should anticholinergic drugs be added to inhaled beta-2 adrenergic agonists in patients with SAE?

R2.3—Inhaled anticholinergic drugs should be combined with beta-2 adrenergic agonists in adult and pediatric patients with SAE.

GRADE 1+, STRONG AGREEMENT

R2.4—The experts suggest administering a 0.5-mg dose of ipratropium bromide every 8 h in adults and children over 6 years of age, and a 0.25-mg dose every 8 h in children under 6 years of age.

EXPERT OPINIONS

#### Rationale

The question of the addition of anticholinergic agents to bronchodilators has been addressed in several studies [30–32] and meta-analyses, including Cochrane studies in adults [33] and children [34]. Compared with the administration of beta-2 adrenergic agonists alone, the anticholinergic/bronchodilator combination increased FEV1 and PEF. A significant decrease of about 30% in hospitalizations was observed. It nonetheless seemed that the benefit of anticholinergics was greater in patients with the lowest PEF on admission. Their use is, therefore, less recommended in patients with non-severe asthma exacerbation. The use of repeated doses of anticholinergic drugs during initial management of SAE patients has not been shown to be beneficial. There was an overall increase in adverse effects frequency (dry mouth, tremor, nausea, headache, agitation) with combination therapy compared with beta-2 adrenergic agonists alone [32], without modification of the benefit–risk ratio. Despite a lack of data, the effect of anticholinergics in SAE seems to be unrelated to dose [35] and a single administration in the acute phase does not seem less effective clinically than a dose repeated every 60 min [33]. The experts propose a dosage regimen of 0.5 mg of ipratropium bromide every 8 h for adults and children over 6 years of age and of 0.25 mg every 8 h for children under 6 years of age.

### Second area: pharmacological treatment

What are the methods of administration of corticosteroid therapy in patients with SAE?

R2.5 adult—Systemic corticosteroid therapy should be administered early intravenously or orally (1 mg/kg of methylprednisolone equivalent, maximum 80 mg per day) to all adult patients with SAE.

GRADE 1+, STRONG AGREEMENT

#### Rationale

A meta-analysis has shown that it is important to administer corticosteroids early, during the first hour of management of patients with SAE [36]. There is no evidence that the intravenous route is superior to the oral route (unless the latter is contraindicated) [36]. Inhalation has not proven better than the intravenous and oral routes and does not seem to be necessary as an adjunct to these two routes [37]. High doses of corticosteroids have not proven superior to low doses. A dose not exceeding 80 mg/day of methylprednisolone or 400 mg of hydrocortisone, therefore, seems sufficient [38].

R2.5 pediatric—Systemic corticosteroid therapy should probably be administered early intravenously or orally (2 mg/kg of methylprednisolone equivalent, maximum 80 mg per day) to children with SAE.

GRADE 2+, STRONG AGREEMENT

#### Rationale

A single randomized, controlled, double-blind pediatric study [39] comparing the use of systemic corticosteroid therapy with inhaled corticosteroid therapy in SAE showed that systemic corticosteroid therapy was more effective in terms of respiratory parameters improvement. Other prospective studies [40–42] have yielded more heterogeneous results, given that they were conducted in smaller study populations or did not specify the severity of asthma exacerbations [43].

### Second area: pharmacological treatment

Is magnesium sulfate indicated in patients with SAE?

R2.6 adult—Magnesium sulfate should probably not be administered routinely to adult patients with SAE.

GRADE 2−, STRONG AGREEMENT

#### Rationale

Intravenous or inhaled magnesium sulfate has been proposed as an adjuvant treatment of SAE because of its interesting experimental properties. However, there is no formal proof of its clinical efficiency. Several studies of low to modest level of proof have yielded discordant results. A well-conducted randomized, controlled study (3-Mg study) [44] showed no difference between asthma patients treated with magnesium sulfate or not regarding the need for hospital admissions, admissions to intensive care, the length of hospitalization, mortality, or the progression of dyspnea or PEF. Benign side effects were significantly more frequent in the magnesium group. It should nonetheless be pointed out that some studies suggest that magnesium sulfate is more effective in the most severely ill patients [15, 45–48], and that there is a certain heterogeneity in the studies regarding the disease severity of included patients. For example, the presence of clinical signs suggestive of a potentially life-threatening condition was an exclusion criterion for the 3 Mg study and resulted in the exclusion of nearly 20% of the eligible patients. A meta-analysis [49] suggested a significant reduction in admissions to intensive care in patients treated with magnesium sulfate, but the heterogeneity of the studies limited the significance of this conclusion. In contrast, the studies were in agreement that this treatment had a relatively good safety profile and was not associated with severe side effects.

R2.6 pediatric—Intravenous magnesium sulfate (dose ≥ 20 mg/kg) should be administered routinely to pediatric patients with SAE.

GRADE 1+, STRONG AGREEMENT

#### Rationale

A meta-analysis of five pediatric studies demonstrated, that in children with moderate to severe asthma exacerbation, magnesium sulfate improved respiratory parameters and reduced both the rate of hospitalization and the use of mechanical ventilation [50], which confirmed the findings of a previous randomized trial [51]. Studies have used different doses and methods of administration, but it seems that a dose equal to or greater than 20 mg/kg is required. In contrast, nebulized magnesium sulfate seems to have no place in the treatment of asthma exacerbation in children [52].

### Second area: pharmacological treatment

Should antibiotic therapy be administered to patients with SAE?

R2.7—Antibiotic therapy should probably not be administered routinely during SAE in adult and pediatric patients. Antibiotic therapy should probably be reserved for cases of suspected bacterial pneumonia, based on usual clinical, radiological, and laboratory signs.

GRADE 2−, STRONG AGREEMENT

#### Rationale

Respiratory infections are a frequent causal factor of SAE. Viruses play a major part, but bacterial infections may also be involved. Whereas the indication for antibiotic therapy is clear in the case of radiological opacity of the lung presumed to be of recent onset, its routine administration in SAE patients is debatable. In conclusion, several arguments may not encourage the routine administration of penicillin or of macrolides to adults with SAE requiring hospitalization [53–56]. Several teams have studied the value of routine antibiotic treatment in SAE requiring hospitalization. In adults, two randomized, controlled trials versus placebo assessed the benefits of routine administration of an oral macrolide for 3–10 days. The endpoint was the regression of respiratory symptoms at day 10. The first trial, which was positive, had several limitations regarding the antibiotic used (telithromycin) and a high rate (61%) of infection by intracellular bacteria in the population of the study [57]. The second trial, using azithromycin, was negative [58]. An older placebo-controlled trial showed no symptomatic benefit of antibiotic therapy with amoxicillin in an adult population [59]. A meta-analysis including trials in children and adults was also negative [60]. In conclusion, there is no argument encouraging the routine administration of penicillin or of macrolides to adults with SAE requiring hospitalization. No study focused specifically on the most severely ill population, i.e., patients with SAE requiring admission to intensive care.

### Third area: methods of oxygen therapy and ventilation

What are the methods of oxygen administration to patients with SAE?

R3.1—Oxygen therapy titrated to a pulse oxygen saturation of 94%–98% should probably be administered to adult and pediatric patients with SAE.

GRADE 2+, STRONG AGREEMENT

#### Rationale

Although patients with SAE are often hypoxemic [61], few studies have evaluated the way of administration of oxygen therapy in these patients: fixed-flow oxygen using a facial mask or administration titrated to pulse oxygen saturation between 94 and 98%. The literature search revealed three studies with a low level of scientific proof (two randomized studies and one observational study) [62–64] that shared numerous biases: difficulties in conducting a blinded study, small study populations, short-term evaluation (20–60 min) and, especially, no evaluation of “hard” outcomes (rates of intubation, intensive care admission, hospitalization, death). A single study observed a deleterious effect of fixed-flow high-concentration oxygen therapy on PEF [63]. In contrast, three studies were in agreement regarding the effect of fixed-flow high-concentration oxygen therapy on the increase in PaCO_2_, compared with titrated oxygen therapy. This outcome alone does not allow a high level of recommendation in the absence of measured clinical impact, in contrast to data on other obstructive lung diseases like chronic obstructive pulmonary disease.

### Third area: methods of oxygen therapy and ventilation

Is there a role for non-invasive ventilation (NIV) or high-flow oxygen therapy in patients with hypoxemic SAE?

R3.2 adult—The experts were unable to recommend the use of NIV in SAE. High-flow nasal oxygen therapy has yet to be assessed in this setting.

EXPERT OPINION

#### Rationale

NIV is increasingly used for the management of SAE [65, 66]. In several observational studies, NIV improved alveolar ventilation and successes were even reported in patients with disturbance of consciousness and with severe hypercapnia. A single study has shown a reduction in the rate of intubation with NIV [67]. However, the conclusions of this retrospective, single-center, before-after study cannot be generalized. In a large American database, the rate of intubation of patients treated by NIV was identical to that of the other patients [68]. Four randomized controlled studies compared NIV with treatment without NIV [69–72]. All these studies reported clinical or spirometric (decrease in respiratory rate, dyspnea, and signs of struggling to breathe, improvement in FEV1 or PEF) improvement. These effects were sometimes accompanied by a reduction in the doses of salbutamol [70] and even in the risk of hospitalization and hospital stay. However, all these studies were in small numbers of patients (30–50). The authors of a systematic Cochrane review concluded that the data were insufficient to assess the effect of NIV on outcomes such as mortality, intubation, improvement in blood gases, and length of hospitalization [73]. In 2017, European and American societies were unable to formulate a recommendation because of the uncertainty and insufficiency of the data [74]. Although NIV seems to have beneficial clinical and spirometric effects, the experts are unable to recommend its use in SAE. No data are yet available regarding high-flow oxygen therapy.

R3.2 pediatric—The use of NIV in children with SAE should probably be considered when conventional treatments fail.

GRADE 2+, STRONG AGREEMENT

R3.2 pediatric—The experts are unable to recommend the use of high-flow nasal oxygen in children with SAE.

EXPERT OPINION

#### Rationale

Three prospective studies, two of which were randomized and controlled [75, 76], found an improvement in clinical parameters and blood gas results when using NIV in SAE [77]. On the other hand, none of these studies showed any benefit in terms of length of hospital stay or the need for invasive ventilation. Data on the use of nasal humidified high-flow oxygen come solely from retrospective studies [78, 79], and so it is currently impossible to recommend its use in clinical practice in children with SAE.

### Third area: methods of oxygen therapy and ventilation

What are the indications for and methods of intubation in patients with SAE?

R3.3—The experts suggest resorting to intubation in adult and pediatric SAE patients if well-conducted medical treatment fails or if the inaugural clinical presentation is severe (altered consciousness, bradypnea). Intubation should be performed using the orotracheal route, after rapid sequence induction including ketamine in first line hypnotic agent and succinylcholine or rocuronium, by an experienced physician.

EXPERT OPINION

#### Rationale

Only 2% of patients hospitalized for SAE require intubation [80]. The usual clinical criteria are the only decision-making arguments for intubation. Given associated high morbidity and mortality rates, intubation will only be considered as a last resort, in case of failure of well-conducted medical treatment or when the clinical presentation is severe at the onset of management (altered consciousness, bradypnea) [81]. It should be performed in accordance with the latest expert guidelines [82] and should be preceded by adequate pre-oxygenation and rapid sequence induction. It should be performed by the most experienced operator so as to reduce the risks of complications [83]. The orotracheal route is preferred. NIV cannot be established as the favored method of pre-oxygenation, but seems logical given the results obtained in other populations of patients. The use of ketamine or propofol as the main hypnotic agent during rapid sequence induction can seem useful because of their theoretical bronchodilator effects [81]. However, no satisfactory scientific data formally validate their use in this indication. As in other indications for emergency intubation, it is justified to use a fast-acting neuromuscular blocker during anesthesia induction [82]. The scientific data concerning the intubation of patients with SAE are extremely limited and come principally from general reviews and expert opinions, or from retrospective cohort studies that are generally single-center and include small numbers of patients. No randomized study, for example, is available regarding methods of intubation. We can, therefore, only formulate an expert opinion.

There is no pediatric study on the indications for intubation in SAE, or on methods of intubation, and practices vary greatly [84]. The indications for intubation should be based on clinical judgment and it is probable that marked hypercapnia or hypoxia, or alterations of consciousness are indications for invasive mechanical ventilation requirement. In addition, a cuffed tracheal tube of the largest possible diameter should probably be preferred.

### Third area: methods of oxygen therapy and ventilation

What are the methods of invasive ventilation in intubated patients with SAE?

R3.4—The experts suggest prevention of lung overdistension by reducing tidal volume, respiratory rate, and positive end-expiratory pressure (PEEP), and by increasing inspiratory flow, to limit plateau pressure in mechanically ventilated adult and pediatric patients with SAE.

EXPERT OPINION

#### Rationale

At the initial phase of management of SAE, lung overdistension by mechanical ventilation is deleterious as it is associated with a risk of barotrauma and induces arterial hypotension [85]. Minute ventilation [65] should be minimized by limiting tidal volume to 6–8 mL/kg [86, 87], o [86], and by increasing inspiratory flow to 60–80 L/min [88]. These goals are attained more easily in controlled volume mode, with constant inspiratory flow [85]. PEEP should be kept ≤ 5 cmH_2_O [89]. Maintenance of plateau pressure < 30 cmH_2_O [13] is associated with a better prognosis. Monitoring of intrinsic PEEP has no benefits [86, 90]. All these settings are generally accompanied by often severe hypercapnia, which should be tolerated (except in the cases of cerebral edema, cranial trauma, and intracerebral mass). It is, therefore, legitimate to prefer a heated humidifier to a heat and moisture exchanger. There are no literature data that allow recommendation of a given value of inspired oxygen fraction (FiO_2_) or an oxygenation target.

No pediatric study has compared one ventilator mode to another for invasive mechanical ventilation in SAE.

### Third area: methods of oxygen therapy and ventilation

What are the methods of sedation for mechanically ventilated patients with SAE? Do halogenated anesthetics have a part to play?

R3.5 adult—The experts suggest deep sedation—Richmond Agitation-Sedation Scale (RASS) of − 4 to − 5—at the initial phase of invasive mechanical ventilation, as well as neuromuscular blockers in the most severely ill patients. Their modalities are not specific to SAE. The experts are not able to recommend continuous administration of ketamine or halogenated agents.

EXPERT OPINION

#### Rationale

Deep sedation (RASS between − 4 and − 5) is often necessary at the initial phase of invasive ventilation given the marked activation of central respiratory drive by the conjunction of the respiratory condition itself, the large reduction in tidal volume, and hypercapnia. For the same reasons, it may be necessary to include continuous or transient neuromuscular block, in the most severely ill patients. Studies in these patients have reported an association between prolonged neuromuscular blockers by atracurium or vecuronium and neuromyopathy acquired in intensive care [91, 92]. This unwanted effect should be taken into account. Among available intravenous sedatives, propofol has more marked bronchodilator properties than benzodiazepines. Because of its own bronchodilator effect, continuously administered ketamine (1–2 mg/kg/h in the adult) in addition to conventional sedation has been evaluated. Studies [93, 94] have reported benefits, at the cost of adverse effects (tachycardia, hallucination/confusion, hypersalivation) that are sometimes severe (myocardial infarction) [95]. Because of their bronchodilator properties, the benefits of halogenated agents (isoflurane and more recently sevoflurane) have been reported, especially in children [96]. An excellent understanding of the pharmacological properties, therapeutic modalities, and side effects of these agents is a necessary prerequisite to their administration.

R3.5 pediatric—Ketamine and halogenated gas should probably not be used for the sedation of mechanically ventilated children with SAE.

GRADE 2−, STRONG AGREEMENT

#### Rationale

A Cochrane meta-analysis [97] suggests that the use of ketamine in children with SAE provides no benefit. There is no other pediatric study on the use of “conventional” sedative agents in children with SAE. The literature on halogenated agents is essentially composed of retrospective studies in small populations and cannot be used to recommend their use in everyday practice [98, 99].

### Third area: methods of oxygen therapy and ventilation

Does helium have a role as carrier gas for inhaled therapies in patients with SAE?

R3.6—Helium should probably not be used as carrier gas in nebulizers in adult and pediatric patients with SAE.

GRADE 2−, STRONG AGREEMENT

#### Rationale

Helium is an inert monoatomic gas whose medical applications are linked to its physical properties and absence of side effects. Compared with an air–oxygen mixture, a helium–oxygen mixture has a lower density and higher viscosity, which improve the transition from turbulent to laminar flow, thus reducing the density-dependent component of bronchial resistance. In SAE, a helium–oxygen mixture optimizes bronchial deposition of bronchodilators. The literature contains 11 prospective randomized trials in adults [100–110] and a meta-analysis [111] comparing nebulization of beta-2 adrenergic agonists (most often albuterol) by a carrier gas composed of a helium–oxygen mixture and a standard mixture of air–oxygen, in patients with SAE. No definitive conclusion can be drawn because these studies were very heterogeneous and included small populations.

### Third area: methods of oxygen therapy and ventilation

What are the methods of nebulization of therapeutic agents for patients with SAE?

R3.7 adult—The experts suggest that aerosols of salbutamol should be administered to spontaneously breathing patients with SAE using a nebulizer. The experts are unable to recommend a particular method of aerosol administration for patients with SAE receiving mechanical ventilation.

EXPERT OPINION

#### Rationale

A meta-analysis showed equivalent efficacy for nebulizers and metered-dose inhalers coupled to valved holding chambers (spacers) [112]. However, in spontaneously breathing patients with SAE, the experts prefer the use of nebulizers, given their greater ease of use and because they do not require the cooperation of the patient [113]. The vast majority of studies have used air- or oxygen-driven pneumatic nebulizers. Oxygen-driven jet nebulization is preferable in the hypoxemic patient, but the expected FiO2 values are low because of the absence of an oxygen reservoir coupled to aerosol masks [114]. With their lower residual volume, ultrasonic nebulizers and vibrating mesh nebulizers have the same efficacy as pneumatic nebulizers and metered dose inhalers used with valved holding chambers [115]. In the absence of clinical evaluation in the setting of asthma, nebulization in a humidified nasal high-flow circuit cannot, at present, be recommended. Placing nebulizers within a high-flow nasal cannula oxygen therapy circuit is associated with a low amount of drug delivered in preclinical studies, and therefore does not seem advisable in the current state of knowledge at the time of guideline writing [116].

The recommendations regarding positive pressure respiratory assistance and high-flow oxygen therapy are only based on expert opinions, in vitro studies, and clinical studies with a high risk of bias in very few subjects. There is no randomized study that has specifically compared different aerosol generators in patients with SAE receiving mechanical ventilation. Ultrasonic nebulizers and vibrating mesh nebulizers and metered-dose inhalers are effective in this context when they can be adapted to the ventilator. There is no reason to alter the ventilator settings because of the administration of aerosols. It is essential to place a filter between the ventilator and the expiratory branch of the respiratory circuit, and to change it regularly [117].

R3.7 pediatric—The experts suggest providing a sufficient flow of air or oxygen to ensure the nebulization of inhaled treatments in spontaneously breathing children with SAE. The experts suggest continuing nebulization using specific systems in children with SAE who are mechanically ventilated.

EXPERT OPINION

#### Rationale

There are no pediatric data on the methods of nebulization in severe acute asthma in children.

### Third area: methods of oxygen therapy and ventilation

What is the role of extracorporeal membrane oxygenation (ECMO) in patients with SAE?

R3.8—In the absence of compelling data in adult and pediatric patients with SAE, the experts suggest discussing with an expert center the use of extracorporeal life support—venovenous ECMO or extracorporeal CO_2_ removal (ECCO2R)—in the case of respiratory acidosis and/or severe hypoxemia refractory to optimal medical treatment and to well-conducted mechanical ventilation.

EXPERT OPINION

#### Rationale

To date, the literature on the use of extracorporeal life support (ECMO or ECCO2R) in the management of SAE is extremely limited. The available data are from small retrospective cohorts [118–121]. Review of the International Extracorporeal Life Support Organization Registry shows that ECMO was used in the setting of SAE in 24 of the 1257 patients included in the registry. Hospital survival was 83%, compared with 51% in patients receiving venovenous ECMO for another cause [121]. As hypercapnia is prominent in refractory SAE, ECCO2R can be considered as a more accessible and less invasive technique than ECMO [118].

The use of extracorporeal life support in children with SAE has been described solely in clinical cases or in retrospective studies in small cohorts [120]. The survival rate in this indication seems good. The criteria for the use of extracorporeal life support have not been described in the literature.

### Fourth area: transfer of patients

What are the criteria allowing hospital discharge of patients with SAE?

R4.1 adult—The experts suggest that the decision to send patients with SAE home should be based on an assessment taking into account the patient’s characteristics, the frequency of exacerbations, the severity of the initial clinical presentation, the response to treatment, including the progression of PEF, and the patient’s ability to be managed at home (referral to the primary care physician).

EXPERT OPINION

#### Rationale

A return home can be envisaged when the symptoms improve after a few hours of treatment in the emergency room. After an hour of continuous treatment with short-acting beta-2 adrenergic agonists, a return home can be envisaged for patients with improved symptoms, no further need for nebulized beta-2 adrenergic agonists, PEF that is 60%–80% of the patient’s theoretical maximum value, pulse oxygen saturation > 94% in ambient air, and a favorable home environment [1]. No study has validated factors predicting readmission of SAE patients.

R4.1 pediatric—The experts are unable to establish pediatric guidelines regarding the decision to send home children admitted for SAE.

EXPERT OPINION

#### Rationale

There are no pediatric data on the subject.

### Fourth area: transfer of patients

For patients treated for SAE, what modalities of hospital discharge from the emergency room reduce the risk of a severe adverse event?

R4.2 adult—The experts suggest that the discharge prescription for patients treated for SAE in the ER should at least include a short-acting beta-2 adrenergic agonist, oral corticosteroid therapy for a short period, and inhaled corticosteroid therapy if it has not been prescribed before.

EXPERT OPINION

#### Rationale

No study has specifically focused on the outcome of patients with SAE not hospitalized after an emergency room stay. On discharge, health professionals must inform the patient and ensure that he or she has a personalized plan of action [1]. A recent meta-analysis did not allow a conclusion to be drawn regarding the impact of these personalized action plans on mortality rate in adults [122]. In contrast, education of the patient reduced the rate of hospitalization in the weeks following the emergency room stay [123]. As for oral corticosteroid therapy, there are no consistent data on the length of treatment, the type of drug or the dose to be preferentially used [124]. A recent Cochrane meta-analysis found no evidence for the additional value of inhaled corticosteroids, alone or combined with oral corticosteroid therapy, upon discharge from the emergency room [37]. However, recent guidelines [1] recommend initiation of inhaled corticosteroids in patients whose chronic treatment does not include them and the increase of their dosage for 2–4 weeks in patients already receiving inhaled corticosteroids. The discharge treatment should also include an inhaled short-acting beta-2 adrenergic agonist and a prescription for PEF device for home monitoring.

R4.2 pediatric—The experts are unable to draw up pediatric guidelines regarding the hospital discharge prescription of children admitted for SAE.

EXPERT OPINION

#### Rationale

There are no pediatric data on the subject.

### Fourth area: transfer of patients

For patients with SAE, what criteria are used to indicate transfer from the emergency room to intensive care?

R4.3—The experts suggest that admission to intensive care of adult and pediatric patients with SAE should be discussed early, on a case-by-case basis, because there are no specific criteria on this subject.

EXPERT OPINION

#### Rationale

No randomized study or large-scale case–control study has conclusively confirmed the criteria justifying intensive care unit admission of patients with SAE. However, epidemiological studies have identified the following epidemiological features correlated with admission to intensive care [68, 125–127]: living in a disadvantaged area, psychiatric illness, substance abuse (heroin, cocaine), poor perception of dyspnea, history of admission to intensive care for SAE, history of intubation for SAE, repeated use of short-acting beta-2 adrenergic agonists, regular use of systemic corticosteroids. The clinical features correlated with admission to intensive care were as follows: clinical signs of respiratory distress, PEF < 200 L/min, improvement in PEF of < 10% after treatment, signs of acute cor pulmonale, poor hemodynamic tolerance, hypercapnia (PaCO_2_ ≥ 45 mmHg) with or without acidemia, metabolic or mixed acidosis, abnormal chest radiography (barotrauma or lung disease). There are no references concerning criteria for the hospitalization of children in continuous monitoring or intensive care pediatric units. Age < 8 years, a history of admission to intensive care for asthma, altered consciousness, and initial severity documented by a clinical score can be used to consider admission to intensive care or to a continuous monitoring unit [128, 129].

### Fifth area: specificities of the pregnant woman

For pregnant women with SAE, does specific management improve morbidity and mortality of mother and fetus compared with standard management?

R5.1—Pregnant women with SAE should probably be treated in the same way as the general population, by intensifying their controlling therapy upon admission to the emergency room if necessary.

GRADE 2+, STRONG AGREEMENT

#### Rationale

There is no study on specific management of SAE in pregnant women. Pregnant women with asthma exacerbation have been shown to receive fewer standard of care therapies in the emergency room [130]. During pregnancy, SAE is frequent and associated with an increased risk of maternal (pre-eclampsia, pre- and post-partum hemorrhage, prelabor rupture of membranes, placental detachment, placenta previa) and fetal and neonatal (growth restriction, hypotrophy, prematurity) complications [131–133]. During SAE, the benefits of treatments are much greater than the very low risk of malformation.

## Data Availability

Not applicable.
